# Changes in HbA1c and weight, and treatment persistence, over the 18 months following initiation of second-line therapy in patients with type 2 diabetes: results from the United Kingdom Clinical Practice Research Datalink

**DOI:** 10.1186/s12916-018-1085-8

**Published:** 2018-07-16

**Authors:** John Wilding, Thomas Godec, Kamlesh Khunti, Stuart Pocock, Robin Fox, Liam Smeeth, Per Clauson, Peter Fenici, Niklas Hammar, Jesús Medina

**Affiliations:** 1 University of Liverpool, Liverpool, UK; 2 London School of Hygiene and Tropical Medicine, London, UK; 3 University of Leicester, Leicester, UK; 4 Bicester Health Centre, Bicester, UK; 5 AstraZeneca, Gothenburg, Sweden; 6 AstraZeneca, Luton, UK; 7 AstraZeneca Farmacéutica Spain, Madrid, Spain

**Keywords:** Clinical practice research datalink (CPRD), Dipeptidyl peptidase-4 inhibitor (DPP-4 inhibitor), HbA1c, Metformin, Second-line therapy, Sodium-glucose cotransporter 2 inhibitor (SGLT-2 inhibitor), Sulfonylurea, Type 2 diabetes (T2D)

## Abstract

**Background:**

Intensification of metformin monotherapy with additional glucose-lowering drugs is often required in patients with type 2 diabetes (T2D). This study evaluated changes in HbA1c and weight, as well as treatment persistence, associated with different second-line therapies used in UK clinical practice.

**Methods:**

The UK Clinical Practice Research Datalink was used to identify patients with T2D who initiated second-line therapy after metformin monotherapy between 1 August 2013 and 14 June 2016. Treatment persistence and changes in HbA1c and weight were assessed at 6-month intervals up to 18 months.

**Results:**

In total, 9097 patients (mean age 61.2 years, 57.2% men, mean [standard deviation] HbA1c 9.0% [1.8]/ 75 mmol/mol [19.7]) were included in the analysis, with a median 2.3 years between initiating metformin monotherapy and initiating second-line therapy. Patients were stratified according to second-line therapy: metformin in combination with sulfonylurea (SU; *n* = 4655 [51.2%]), a dipeptidyl peptidase-4 inhibitor (DPP-4 inhibitor; *n* = 2899 [31.9%]), or a sodium–glucose cotransporter-2 inhibitor (SGLT-2 inhibitor; *n* = 441 [4.9%]) or other therapies (all other second-line treatments; *n* = 1102 [12.1%]). At 18 months, the cumulative proportion of patients changing treatment was lowest for those who received metformin plus an SGLT-2 inhibitor (42.3%), followed by patients on metformin plus SU or metformin plus a DPP-4 inhibitor (46.8%). HbA1c reductions were seen with all second-line therapies, with an overall mean (standard error) reduction of −1.23% (0.05)/−13.4 mmol/mol (0.5). Changes were directly, but not linearly, related to baseline HbA1c and were greater in those with higher HbA1c at baseline. Weight loss from baseline was greatest in patients treated with metformin plus either an SGLT-2 inhibitor (−4.2 kg) or a DPP-4 inhibitor (−1.5 kg). The highest proportion of patients who achieved the composite outcome of HbA1c reduction ≥ 0.5%, body weight loss ≥ 2.0 kg and treatment persistence for 18 months was observed in those receiving metformin plus an SGLT-2 inhibitor (36.5%).

**Conclusions:**

In this population-based cohort, all second-line therapies added to metformin monotherapy improved glycaemic control, but the lowest treatment change/discontinuation rate and most sustained weight loss was seen with patients receiving metformin plus an SGLT-2 inhibitor.

**Electronic supplementary material:**

The online version of this article (10.1186/s12916-018-1085-8) contains supplementary material, which is available to authorized users.

## Background

Type 2 diabetes (T2D) is a complex chronic condition characterised by increased blood glucose levels and associated with micro- and macrovascular complications. In 2015, an estimated 415 million people globally had diabetes, with the vast majority (~ 90%) having T2D [1]. Optimal glycaemic control (i.e. attaining recommended treatment targets) has been shown to reduce the incidence of diabetes-related complications, microvascular disease and myocardial infarction in the large, long-term United Kingdom Prospective Diabetes Study [2, 3].

Along with lifestyle modifications, current guidelines recommend the use of metformin as the preferred first-line glucose-lowering therapy in patients with T2D [4]. Metformin has a low risk of hypoglycaemia and is weight neutral, with no increased risk of or benefit against adverse cardiovascular events [5, 6]. However, in view of its progressive nature, the majority of patients with T2D require treatment intensification from metformin monotherapy to achieve and maintain recommended HbA1c targets [6]. Second-line agents approved for dual therapy with metformin in the UK include sulfonylureas (SUs), thiazolidinediones (TZDs), dipeptidyl peptidase-4 (DPP-4) inhibitors, sodium–glucose cotransporter-2 (SGLT-2) inhibitors, glucagon-like peptide-1 (GLP-1) receptor agonists and basal insulin analogues. The National Institute for Health and Care Excellence (NICE) guidelines and the position statement of the American Diabetes Association and the European Association for the Study of Diabetes recommend that initial treatment intensification after failure of metformin monotherapy should consist of dual therapy with one of the following agents: DPP-4 inhibitors, pioglitazone, SUs or SGLT-2 inhibitors [4, 6]. The choice of second-line agent should be based upon patient-specific considerations to minimise side effects whilst reducing HbA1c levels [7].

Established agents such as SUs are highly efficacious but are associated with weight gain and may cause hypoglycaemia [8]. TZDs do not induce hypoglycaemia, but tend to cause weight gain and fluid retention, and have been associated with an increased risk of heart failure [6, 9]. Unlike SUs and TZDs, DPP-4 inhibitors do not lead to weight gain and a have minimal risk for hypoglycaemia, comparable to metformin [10, 11]. Likewise, SGLT-2 inhibitors do not induce hypoglycaemia and bring the added benefits of reductions in body weight and blood pressure [12–14]. Results from the EMPA-REG OUTCOME [15] and CANVAS [16] randomised controlled trials and the CVD-REAL observational studies [17–19] have shown a reduced risk for major adverse cardiovascular events, hospitalisation for heart failure or death in patients with T2D treated with SGLT-2 inhibitors, with additional studies ongoing [20]. GLP-1 receptor agonists are associated with low risk for hypoglycaemia and significant reductions in body weight [21]. GLP-1 receptor agonists are not generally recommended as a second-line therapy in the UK [22], and their effect on adverse cardiovascular outcomes is inconsistent. While liraglutide has been associated with a reduction in adverse cardiovascular outcomes in patients with T2D at high cardiovascular risk, a neutral effect on cardiovascular outcomes has been observed with other GLP-1 receptor agonists [23–26]. Of all the glucose-lowering agents, insulin has the greatest HbA1c-lowering potential, although it carries the highest risk of hypoglycaemia and is associated with weight gain [6, 21].

The NICE guidelines and the position statement of the American Diabetes Association and the European Association for the Study of Diabetes recommend that therapies should be reviewed every 3–6 months, and therapy escalated if HbA1c targets are not met. Despite these recommendations, therapeutic inertia is still common, with the average time to initiate second-line therapy in the UK being between 1.6 to 2.9 years after HbA1c levels had reached those recommended for treatment intensification (HbA1c > 7.5% [58 mmol/mol]) [27]. Likewise, in patients from North America, Europe and Israel receiving one oral anti-diabetic drug, second-line treatment intensification occurs a median of 0.3–2.7 years following above-target HbA1c levels [28]. This may carry grave consequences for patients who have poor glycaemic control for extended periods and may result in higher risk of cardiovascular events and other late-stage diabetes complications [29]. On the other hand, patients who receive earlier treatment intensification have greater HbA1c reductions and a higher probability of attaining target HbA1c levels, as well as a lower risk of micro- and macrovascular complications, compared with patients with delayed intensification [2, 3, 30–32].

Real-world clinical-practice data are an important source of evidence for clinicians considering second-line therapies for the treatment of T2D. This study assessed the clinical outcomes of patients initiated on second-line glucose-lowering therapies, using data from the UK Clinical Practice Research Datalink (CPRD). The impact of these second-line therapies on HbA1c, weight and treatment persistence was evaluated over 18 months in patients who had received first-line metformin monotherapy. The data in this manuscript were accepted as an abstract for - and presented at - the 53rd Annual Meeting of the European Association for the Study of Diabetes [33].

## Methods

### Data source

This retrospective study was performed using data retrieved from the UK CPRD, which contains anonymised longitudinal primary-care medical records from over 14 million patients and is broadly representative of the UK population [34]. The study protocol was approved by the independent scientific advisory committee of the UK CPRD (protocol 16_045R).

### Study population

The cohort comprised patients with T2D aged ≥ 18 years who initiated a second-line therapy between 1 August 2013 and 14 June 2016. Only patients who received metformin monotherapy as first-line therapy were included in the analysis, irrespective of the duration of metformin treatment before initiation of second-line therapy and HbA1c values at the time of treatment initiation. Patients were grouped according to second-line therapy, which could include any glucose-lowering medication, oral or injectable, given either as an add-on to metformin or as a switch to a single agent or in combination with other agents after discontinuing metformin. For this analysis, patients were stratified into one of the following groups: metformin in combination with an SU (glibenclamide, glipizide, gliclazide, glimepiride, tolbutamide or chlorpropamide), metformin in combination with a DPP-4 inhibitor (sitagliptin, vildagliptin, saxagliptin, linagliptin or alogliptin), metformin in combination with an SGLT-2 inhibitor (canagliflozin, dapagliflozin or empagliflozin), or metformin in combination with any other second-line therapy (including pioglitazone, a GLP-1 receptor agonist, an insulin, repaglinide, natiglinide, acarbose) or another second-line therapy following metformin discontinuation.

### Study design

This was an observational study comparing the use of second-line glucose-lowering agents in patients with T2D who received metformin as their first-line therapy. The index date was the date of second-line therapy initiation, and baseline measurements of HbA1c and weight closest to the index date (within 180 days prior to the index date and 2 weeks after the index date) were selected for analysis. The follow-up periods were defined as 6, 12 and 18 months, with measurements closest to each time point (±90 days) selected for the analysis. To represent an on-treatment population, data were analysed for patients who were receiving their index second-line treatment at each time point (i.e. had not intensified, switched or discontinued treatment) and who had data available for the variable of interest (HbA1c or weight) at baseline and the time point being analysed. Discontinuation of second-line therapy was defined as cessation of treatment for 184 days or more.

### Study outcomes

Index treatment persistence over the 18-month study period was evaluated. HbA1c and weight were evaluated for all patients who were on-treatment and had data available at both baseline and the end of the corresponding follow-up period. A composite outcome assessed treatment success, defined as the number of patients who had not discontinued their index therapy, achieved HbA1c reductions ≥ 0.5% (5.5 mmol/mol) and weight reductions ≥ 2 kg at 18 months. The individual components of the composite endpoint were also evaluated for all patients who had HbA1c and weight data available at baseline and at 18 months.

### Statistical analysis

The characteristics of the study population were summarised with descriptive statistics (number, mean, standard deviation [SD], standard error [SE], median and interquartile range [IQR]). Demographics and baseline characteristics, as well as changes in HbA1c and weight over time, are presented separately for the different second-line treatment groups. Mean (SE) HbA1c and weight at baseline and at the 6-, 12- and 18-month time points were used to calculate crude changes over each 6-month period. For each second-line therapy group, the change in HbA1c and weight at each time point was determined using analysis of covariance (ANCOVA) models, adjusted for baseline measurements using variance-weighted least-squares regression estimation.

The relationship between baseline measurements and the changes at each subsequent time point was explored to determine the best way to adjust for baseline measurements. The relationship between baseline HbA1c and change in HbA1c was not strictly linear and was modelled using linear splines as two straight lines connected at a baseline HbA1c of 9% (75 mmol/mol) [28]. The relationship between baseline body mass index (BMI) and change in BMI showed no evidence of departure from linearity and was, therefore, modelled in a linear manner.

The composite outcome (number [%] of patients who had not discontinued index therapy, achieved HbA1c reductions of ≥ 0.5% [5.5 mmol/mol] and weight reductions of ≥ 2 kg at 18 months) was tabulated by second-line treatment type.

## Results

### Patient populations

From the initial 407,700 patients screened, we identified 9097 patients with T2D who had received first-line metformin monotherapy and were initiated on second-line treatment (Additional file 1: Figure S1). The median (IQR) time between initiating metformin and initiating second-line therapy was 2.3 (0.85–4.72) years. The most common second-line treatments were metformin plus an SU (*n* = 4655; 51.2%), metformin plus a DPP-4 inhibitor (*n* = 2899; 31.9%) and metformin plus a SGLT-2 inhibitor (*n* = 441; 4.8%). The remaining 1102 patients (12.1%) were on a variety of second-line treatments and were combined into the other category. This included monotherapy with an SU (*n* = 397; 4.4%), monotherapy with a DPP-4 inhibitor (*n* = 212; 2.3%), monotherapy with any other agent (*n* = 38; 0.4%), insulin either as mono- or combination therapy (*n* = 46; 0.5%) or any other drug combinations not specified above (*n* = 409; 4.5%).

The baseline characteristics of the patients included in the analysis are shown in Table 1. Overall 57.2% of the patients were male, their mean age was 61.2 years and the mean HbA1c was 9.0% (75 mmol/mol). The baseline characteristics varied among the groups, with higher mean baseline HbA1c values observed for patients who received metformin plus an SU or metformin plus an SGLT-2 inhibitor, compared with patients who received metformin plus a DPP-4 inhibitor or other therapies. Patients who were initiated on second-line therapy with an SGLT-2 inhibitor had the highest mean BMI and body weight values, with patients who were initiated on second-line therapy with a DPP-4 inhibitor having the second highest values. Furthermore, patients who were initiated on second-line treatment with an SGLT-2 inhibitor were younger and had the shortest duration between initiation of metformin and initiation of second-line therapy. Patients in the other therapy group had the longest interval between initiation of first- and second-line therapies.

**Table 1:** Demographic and baseline characteristics

	MET + SU *N* = 4655	MET + DPP-4i *N* = 2899	MET + SGLT-2i *N* = 441	Other* N = 1102	Overall *N* = 9097
Male, *n* (%)	2679 (57.6)	1704 (58.8)	266 (60.3)	551 (50.0)	5200 (57.2)
Age (years), mean (SD)	61.54 (12.97)	60.81 (12.34)	55.05 (10.14)	62.81 (14.85)	61.15 (12.98)
<50	892 (19.2%)	544 (18.8%)	128 (29.0%)	210 (19.1%)	1774 (19.5%)
50 to <60	1150 (24.7%)	805 (27.8%)	165 (37.4%)	241 (21.9%)	2361 (26.0%)
60 to <70	1291 (27.7%)	842 (29.0%)	119 (27.0%)	275 (25.0%)	2527 (27.8%)
≥70	1322 (28.4%)	708 (24.4%)	29 (6.6%)	376 (34.1%)	2435 (26.8%)
HbA1c (%), mean (SD)	9.19 (1.88)	8.75 (1.50)	9.07 (1.58)	8.72 (1.91)	8.98 (1.77)^†^
<7	283 (6.2%)	147 (5.1%)	20 (4.6%)	164 (15.2%)	614 (6.9%)
7 to <8	971 (21.3%)	829 (28.8%)	100 (23.0%)	276 (25.5%)	2176 (24.3%)
8 to <9	1245 (27.3%)	923 (32.1%)	120 (27.7%)	254 (23.5%)	2542 (28.4%)
9 to <10	783 (17.2%)	468 (16.3%)	84 (19.4%)	152 (14.1%)	1487 (16.6%)
10 to <11	491 (10.8%)	244 (8.5%)	54 (12.4%)	92 (8.5%)	881 (9.8%)
≥11	787 (17.3%)	266 (9.3%)	56 (12.9%)	144 (13.3%)	1253 (14.0%)
HbA1c (mmol/mol), mean (SD)	77 (20.6)	72 (16.4)	76 (17.3)	72 (20.9)	75 (19.3)^†^
BMI (kg/m^2^, mean (SD)	31.78 (6.50)	33.21 (6.56)	36.56 (6.98)	33.12 (7.93)	32.64 (6.82)^†^
<20	43 (0.9%)	8 (0.3%)	0	24 (2.2%)	75 (0.8%)
20 to <25	498 (10.9%)	194 (6.8%)	8 (1.8%)	106 (9.8%)	806 (9.0%)
25 to <30	1457 (31.9%)	795 (27.7%)	65 (14.8%)	273 (25.2%)	2590 (28.9%)
30 to <35	1366 (29.9%)	887 (31.0%)	129 (29.5%)	301 (27.8%)	2683 (30.0%)
≥35	1208 (26.4%)	982 (34.3%)	236 (53.9%)	379 (35.0%)	2805 (31.3%)
Weight (kg), mean (SD)	90.97 (21.00)	96.00 (21.50)	107.30 (22.87)	93.63 (25.55)	93.70 (22.18)^†^
<60	189 (4.1%)	63 (2.2%)	2 (0.5%)	51 (4.7%)	305 (3.4%)
60 to <80	1227 (26.6%)	578 (20.1%)	40 (9.1%)	298 (27.3%)	2143 (23.8%)
80 to <100	1837 (39.9%)	1133 (39.3%)	127 (28.9%)	361 (33.1%)	3458 (38.4%)
100 to <120	954 (20.7%)	728 (25.3%)	166 (37.7%)	232 (21.3%)	2080 (23.1%)
≥120	399 (8.7%)	378 (13.1%)	105 (23.9%)	149 (13.7%)	1031 (11.4%)
Time since diagnosis (years), median (IQR)	4.00 (1.57–7.20)	4.25 (1.88–7.21)	3.30 (1.47–5.55)	4.64 (1.94–8.05)	4.10 (1.70–7.22)
<6 months	542 (11.6%)	237 (8.2%)	40 (9.1%)	74 (6.7%)	893 (9.8%)
6 months to <1 year	283 (6.1%)	174 (6.0%)	35 (7.9%)	68 (6.2%)	560 (6.2%)
1 to <3 years	1076 (23.1%)	658 (22.7%)	129 (29.3%)	250 (22.7%)	2113 (23.2%)
3 to <5 years	839 (18.0%)	591 (20.4%)	104 (23.6%)	194 (17.6%)	1728 (19.0%)
≥5 years	1915 (41.1%)	1239 (42.7%)	133 (30.2%)	516 (46.8%)	3803 (41.8%)
Time since initiation of first-line therapy (years), median (IQR)	2.20 (0.72–4.65)	2.62 (1.02–4.89)	2.05 (0.85–4.02)	2.30 (0.89–4.84)	2.33 (0.85–4.72)
<6 months	953 (20.5%)	427 (14.7%)	71 (16.1%)	171 (15.5%)	1622 (17.8%)
6 months to <1 year	474 (10.2%)	280 (9.7%)	50 (11.3%)	124 (11.3%)	928 (10.2%)
1 to <3 years	1346 (28.9%)	884 (30.5%)	157 (35.6%)	346 (31.4%)	2733 (30.0%)
3 to <5 years	840 (18.1%)	613 (21.2%)	89 (20.2%)	199 (18.1%)	1741 (19.1%)
≥5 years	1042 (22.4%)	695 (24.0%)	74 (16.8%)	262 (23.8%)	2073 (22.8%)

*BMI* body mass index, *DPP-4i* dipeptidyl peptidase-4 inhibitor, *HbA1c* glycated haemoglobin, *IQR* interquartile range, *MET* metformin, *SD* standard deviation, *SGLT-2i* sodium–glucose cotransporter 2 inhibitor, *SU* sulfonylurea

^*^Includes all other add-on combinations to metformin, combinations without metformin and other monotherapies

^†^Baseline HbA1c, BMI and weight were missing for 144, 138 and 80 patients, respectively

### Persistence of second-line treatment

**Fig. 1:**
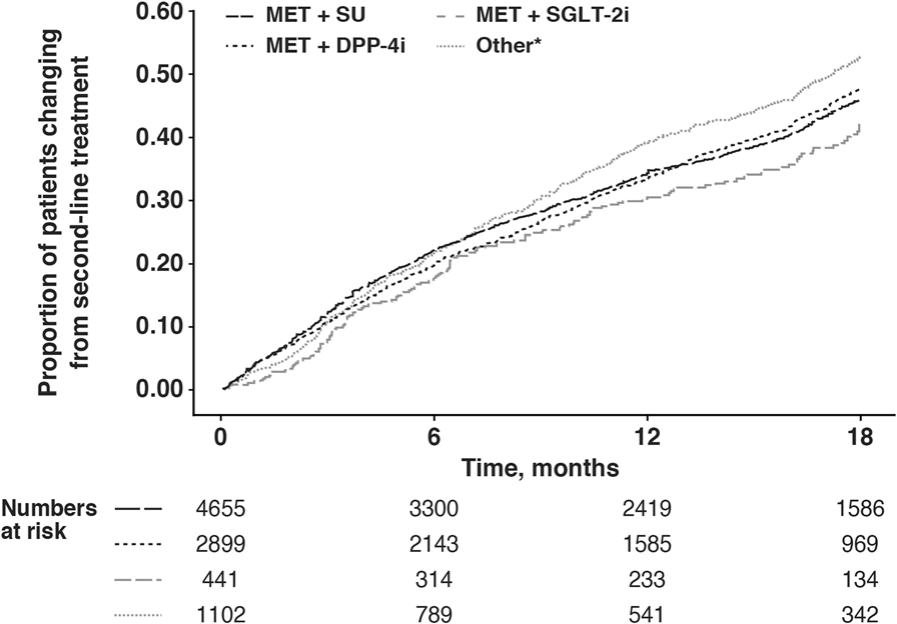
Cumulative proportion of patients changing second-line therapy over time according to treatment group. Frequency is shown as proportion of 1. DPP-4i dipeptidyl peptidase-4 inhibitor, MET metformin, SGLT-2i sodium–glucose cotransporter 2 inhibitor, SU sulfonylurea. *Includes all other add-on combinations to metformin, combinations without metformin and other monotherapies

The proportion of patients who changed or discontinued their index second-line therapy over the 18-month follow-up period for each treatment group is shown in Fig. 1. Overall, the cumulative proportion changing therapy (including discontinuation, switch or intensification) was 21.6%, 34.8% and 47.3% at 6, 12 and 18 months of follow-up, respectively.

Across all time points, fewer patients receiving metformin plus an SGLT-2 inhibitor changed therapy, with 42.3% cumulative proportion of patients changing treatment by 18 months. For patients receiving metformin plus either an SU or a DPP-4 inhibitor, the cumulative proportion of patients changing treatment for both therapies was 46.8%. Patients receiving other regimens changed treatment most frequently, with 52.8% cumulative proportion of patients changing treatment at 18 months, a significantly higher incidence compared with that seen in patients receiving metformin plus either an SU or a DPP-4 inhibitor (*p* = 0.003).

### Changes in HbA1c

For all patients initiated on second-line therapy (including those who subsequently discontinued, switched or intensified treatment), the mean unadjusted changes in HbA1c were −1.26% (−13.8 mmol/mol; *n* = 6042), −1.16% (−12.7 mmol/mol; *n* = 4945) and −0.99% (−10.8 mmol/mol; *n* = 3665) at 6, 12 and 18 months, respectively. When only patients who were on-treatment (i.e. had not intensified, switched or discontinued second-line treatment) were included in the analysis, the mean unadjusted changes in HbA1c were −1.38% (−15.1 mmol/mol; *n* = 4480), −1.32% (−14.4 mmol/mol; *n* = 3080) and − 1.20% (−13.1 mmol/mol; *n* = 1755) at 6, 12 and 18 months, respectively.

**Fig. 2:**
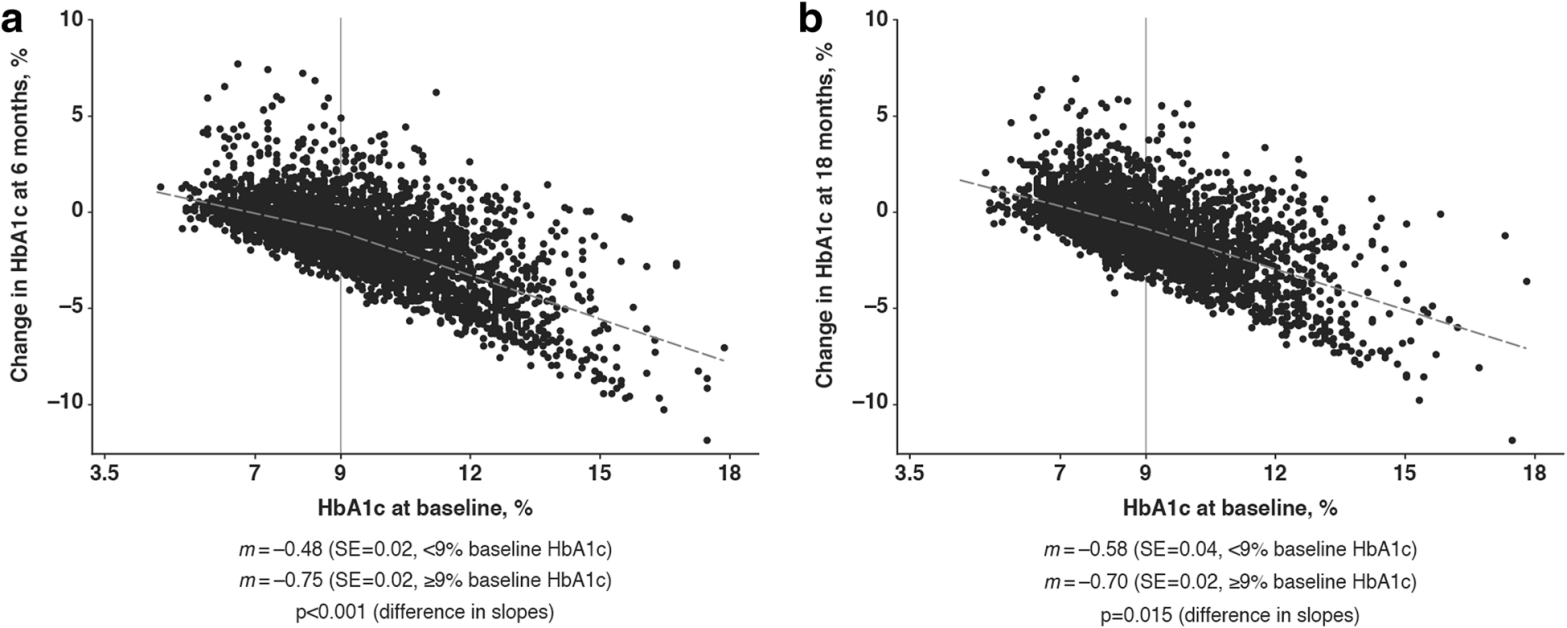
Relationship between change in HbA1c and baseline HbA1c (patient-level data). Scatter plots of baseline (index) HbA1c against change in HbA1c at **a** 6 and **b** 18 months. Regression lines are shown as grey dashed lines, and their corresponding gradient slopes (*m*) were calculated for baseline HbA1c levels < 9% and ≥ 9%. SE standard error

Analysis of HbA1c levels at 6 months shows that patients with lower baseline HbA1c levels experienced smaller changes from baseline, and fewer achieved HbA1c reductions ≥ 0.5% (5.5 mmol/mol) compared with patients with a higher baseline HbA1c (Table 2). The change in HbA1c levels at 6 and 18 months correlated with baseline HbA1c levels, and the changes were more marked in patients with baseline HbA1c levels ≥ 9.0% (75 mmol/mol; *p* < 0.001; Fig. 2). At 6 months, patients who had baseline HbA1c levels < 9.0% (75 mmol/mol), had an expected additional HbA1c decrease of 0.48% (5.2 mmol/mol) for every 1.0% (10.9 mmol/mol) increment in baseline HbA1c, whereas patients with baseline HbA1c levels ≥ 9.0% had an expected additional HbA1c decrease of 0.75% (8.2 mmol/mol) for every 1.0% increment. This correlation was maintained throughout the 18 months of therapy (*p* = 0.015).

**Table 2:** Change in HbA1c at 6 months post-initiation of second-line treatment, stratified by baseline HbA1c category

Baseline HbA1c		*N*	Mean change at 6 months (standard error)		*N* (%) with fall ≥ 0.5% at 6 months
%	mmol/mol		%	mmol/mol	
<7.5	< 58	811	0.02 (0.04)	0.2 (0.4)	277 (34.2)
7.5 to <9.0	58 to <75	2751	−0.71 (0.02)	−7.8 (0.2)	1855 (67.4)
9.0 to <10.5	75 to <91	1325	−1.47 (0.04)	−16.1 (0.4)	1099 (82.9)
10.5 to <12.0	91 to <108	715	−2.48 (0.06)	−27.1 (0.7)	641 (89.7)
≥12.0	≥108	440	−4.40 (0.12)	−48.1 (1.3)	405 (92.1)
Overall		6042	−1.26 (0.02)	−13.8 (0.2)	4277 (70.8)

Data from patients who had available HbA1c measurements at 6 months and at baseline, and who were on-treatment at 6 months (i.e. had not switched, intensified or discontinued second-line treatment since the previous time point)

The mean changes in HbA1c over 18 months, after adjustment for baseline values for each second-line treatment group, are shown in Table 3. At 6 months, patients receiving metformin plus an SU or metformin plus an SGLT-2 inhibitor had the greatest mean reductions in HbA1c (−1.33% [−14.5 mmol/mol] and − 1.26% [−13.8 mmol/mol], respectively), and, combined, these reductions were significantly greater (*p* < 0.001) than those observed in patients receiving metformin plus a DPP-4 inhibitor or other therapies (−1.11% [−12.1 mmol/mol] and − 1.03% [−11.3 mmol/mol], respectively). At 18 months, there was a trend for patients receiving metformin plus an SGLT-2 inhibitor to achieve greater mean reductions in HbA1c, compared with patients receiving any other treatment (−1.46% [−16 mmol/mol] versus −1.21% [−13.2 mmol/mol]; *p* = 0.052; Table 3).

**Table 3:** Change in HbA1c levels over 18 months after initiation of second-line treatment

Second-line treatment	6 months			12 months			18 months		
	*N* (%)	Mean change in HbA1c (SE)*		*N* (%)	Mean change in HbA1c (SE)*		*N* (%)	Mean change in HbA1c (SE)*	
		%	mmol/mol		%	mmol/mol		%	mmol/mol
MET + SU	2257 (50.38)	−1.33 (0.04)	−14.5 (0.4)	1571 (51.01)	−1.30 (0.04)	−14.2 (0.4)	907 (51.68)	−1.21 (0.05)	−13.2 (0.5)
MET + DPP-4i	1486 (33.17)	−1.11 (0.04)	−12.1 (0.4)	1044 (33.90)	−1.12 (0.05)	−12.2 (0.5)	572 (32.59)	−1.23 (0.06)	−13.4 (0.7)
MET + SGLT-2i	225 (5.02)	−1.26 (0.08)	−13.8 (0.9)	156 (5.06)	−1.32 (0.10)	−14.4 (1.1)	90 (5.13)	−1.46 (0.13)	−16.0 (1.4)
Other^†^	512 (11.43)	−1.03 (0.06)	−11.3 (0.7)	309 (10.03)	−1.20 (0.08)	−13.1 (0.9)	186 (10.60)	−1.18 (0.10)	−12.9 (1.1)
Overall	4480	−1.23 (0.03)	−13.4 (0.3)	3080	−1.24 (0.04)	−13.6 (0.4)	1755	−1.23 (0.05)	−13.4 (0.5)

Data from patients who had available HbA1c measurements at each time point and at baseline, and who were on-treatment at each time point (i.e. had not switched, intensified or discontinued second-line treatment since the previous time point)

*Baseline adjusted means using analysis of covariance

^†^Includes all other add-on combinations to metformin, combinations without metformin and other monotherapies

*DPP-4i* dipeptidyl peptidase-4 inhibitor, *MET* metformin, *SGLT-2i* sodium–glucose cotransporter 2 inhibitor, *SE* standard error, *SU* sulfonylurea

### Changes in weight

**Fig. 3:**
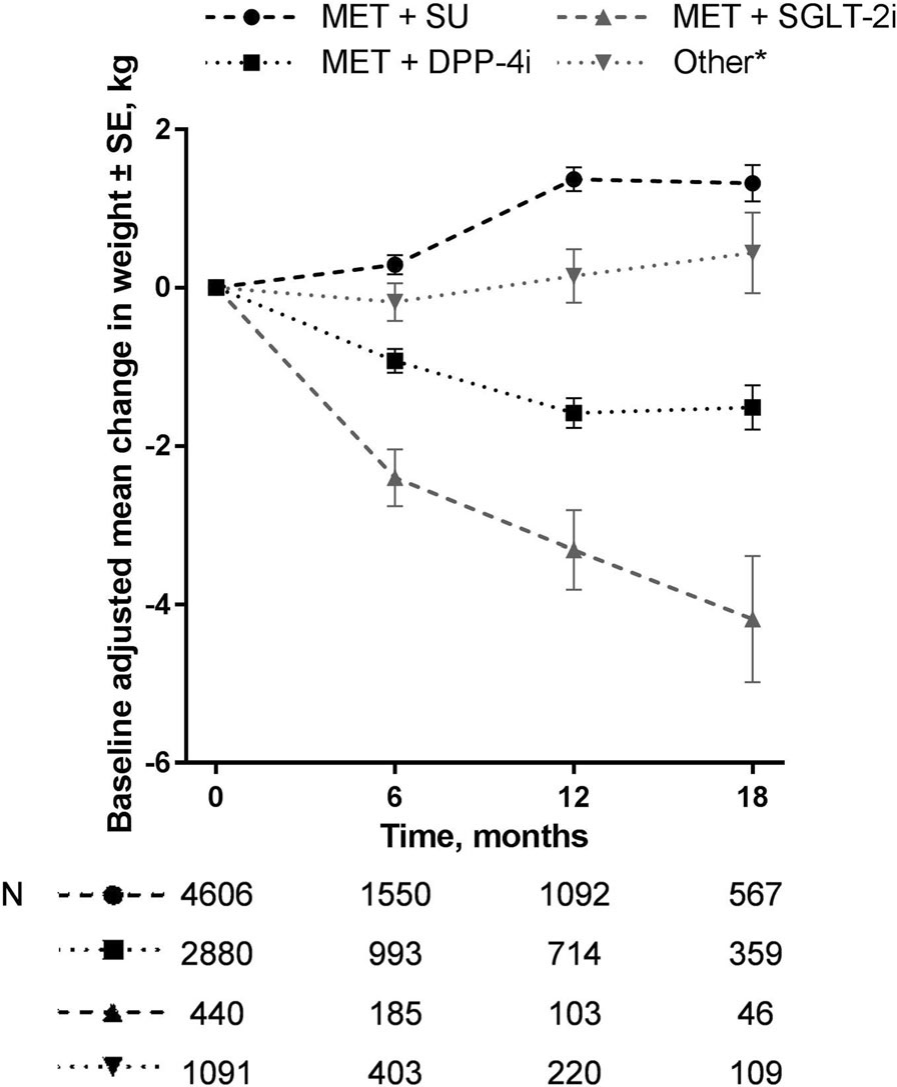
Baseline-adjusted change in weight over 18 months in patients initiated on second-line therapies. Data are means ± SE. Data are from patients who had available weight measurements at each timepoint and at baseline, and who were on-treatment at each time-point (i.e. had not switched, intensified or ceased second-line treatment since the previous time-point). DPP-4i, dipeptidyl peptidase-4 inhibitor; MET, metformin; N, number of patients; SGLT-2i, sodium–glucose cotransporter 2 inhibitor; SE, standard error; SU, sulfonylurea. *Includes all other add-on combinations to metformin, combinations without metformin and other monotherapies

In all four treatment groups, weight loss from baseline at 18 months was directly correlated with baseline weight (Additional file 2: Figure S2). The mean baseline-adjusted changes in weight at 6, 12 and 18 months for each treatment group are shown in Fig. 3. The greatest mean reduction in weight from baseline was observed for patients who received metformin plus an SGLT-2 inhibitor (mean weight loss at 18 months, 4.2 kg; *p* < 0.001 versus baseline). A significant mean reduction in weight from baseline was also observed for patients receiving metformin plus a DPP-4 inhibitor (mean weight loss at 18 months, 1.5 kg; *p* < 0.001 versus baseline). In contrast, small but significant mean increases in weight from baseline (*p* < 0.001) were observed at all time points for patients receiving metformin plus an SU. Overall, no pattern of weight change was observed for patients receiving other second-line treatments.

### Achievement of the composite outcome of treatment success according to second-line therapy

To evaluate treatment success, a composite outcome, defined as the number of patients who had not discontinued their index therapy, achieved HbA1c reductions ≥0.5% (5.5 mmol/mol) and weight reductions ≥ 2 kg at 18 months, was assessed for all patients with data available at baseline and 18 months (Table 4). A higher proportion of patients assigned to the metformin plus an SGLT-2 inhibitor treatment group achieved HbA1c reductions ≥0.5% (71.6%) or weight reductions ≥ 2.0 kg (71.6%) at 18 months, compared with all the other treatment groups analysed. Of patients who intensified metformin treatment with a DPP-4 inhibitor, 55.1% achieved HbA1c reductions ≥ 0.5% and 42.8% of patients had weight reductions ≥ 2.0 kg. By comparison, 66% of patients on metformin plus an SU group achieved HbA1c reductions ≥ 0.5%, although only 25.6% achieved weight loss ≥ 2.0 kg at 18 months. Treatment persistence at 18 months was highest for patients receiving metformin plus an SU (56.4%) and metformin plus an SGLT-2 inhibitor (55.4%).

**Table 4:** Composite outcome representing treatment success after 18 months for each treatment group

Second-line treatment	*N*	HbA1c reduction ≥ 0.5% (≥ 5.5 mmol/mol)	Weight-loss ≥ 2 kg	No treatment change over 18 months	Overall success (i.e. attained all three endpoints)
MET + SU	847	559 (66.0)	217 (25.6)	478 (56.4)	81 (9.6)
MET + DPP-4i	608	335 (55.1)	260 (42.8)	300 (49.3)	104 (17.1)
MET + SGLT-2i	74	53 (71.6)	53 (71.6)	41 (55.4)	27 (36.5)
Other*	187	105 (56.2)	61 (32.6)	91 (48.7)	17 (9.1)
Overall	1716	1052 (61.3)	591 (34.4)	910 (53.0)	229 (13.3)

Data are in *n* (%). Data from all patients who had both HbA1c and weight measurements available at baseline and at 18 months

*DPP-4i* dipeptidyl peptidase-4 inhibitor, *MET* metformin, *SGLT-2i* sodium–glucose cotransporter 2 inhibitor, *SU* sulfonylurea

*Includes all other add-on combinations to metformin, combinations without metformin and other monotherapies

Overall, 36.5% of patients receiving metformin plus an SGLT-2 inhibitor met the composite outcome for treatment success, compared with 17.1% of those who received metformin plus a DPP-4 inhibitor, 9.6% of those who received metformin plus an SU and 9.1% of patients who received other treatments. This pattern was confirmed after adjustment for baseline HbA1c, weight, age, sex and time since initiation of first-line treatment. Multivariate analysis showed that initiation of second-line therapy with an SGLT-2 inhibitor resulted in a significantly higher overall success rate, compared with all other second-line therapies combined (*p* < 0.001).

## Discussion

This study used a large, representative population of UK patients to evaluate the use of second-line therapies after metformin monotherapy in a real-world clinical practice setting.

Unlike most clinical trials, which have durations of 6–12 months, this observational study followed patients for 18 months, with 6-month follow-up intervals, and compared the effect of initiation of different second-line therapies on three clinically relevant criteria: changes in HbA1c and weight, and treatment persistence. When these three criteria were evaluated as a composite outcome representative of treatment success at 18 months, more patients (36.5%) on metformin plus an SGLT-2 inhibitor achieved HbA1c reductions of ≥ 0.5% (5.5 mmol/mol), body weight loss ≥ 2 kg and treatment persistence for 18 months, compared with all other therapies. Patients who received combination therapy with metformin plus a DPP-4 inhibitor achieved the second highest composite outcome, with 17.1% of patients achieving treatment success.

In this study, all second-line therapies reduced HbA1c levels, with a mean reduction of 1.2% (13.4 mmol/mol) at 18 months. HbA1c reductions achieved at 6 months following second-line treatment initiation correlated with HbA1c levels at baseline: patients with lower baseline levels experience smaller HbA1c reductions than patients who had higher baseline levels. This observation is in line with previous studies, which report a positive correlation between baseline HbA1c and subsequent changes in HbA1c levels [28, 35]. The greatest reductions in HbA1c at 6 months were observed in patients on metformin plus an SU or on metformin plus an SGLT-2 inhibitor, although at 18 months, these reductions were observed only in patients on metformin plus an SGLT-2 inhibitor. It is known that SUs are highly efficient at rapidly reducing HbA1c levels in patients with T2D, with only insulin acting on HbA1c faster [6]. However, many clinical trials comparing SU to other therapies have shown the effects of SUs on HbA1c to be short-lived [36–38]. The sustained reduction in HbA1c levels observed in patients receiving metformin plus an SGLT-2 inhibitor has been previously reported in a meta-analysis [39]. However, the higher HbA1c levels at baseline and the reduced number of patients who had HbA1c measurements at 18 months in the CPRD database may have contributed to the effect seen.

The effect of a particular treatment on weight is also an important factor when deciding upon an appropriate second-line agent, as obesity is often associated with T2D [40]. Overall, modest weight gain was seen in patients who received the combination of metformin plus an SU, whereas weight loss was observed for patients treated with metformin plus either a DPP-4 inhibitor or an SGLT-2 inhibitor, with significantly greater weight loss observed in patients receiving an SGLT-2 inhibitor. While DPP-4 inhibitors are generally considered to be weight neutral [6], reductions in BMI following treatment with a DPP-4 inhibitor have been observed in a previous database analysis in Italy [41]. Weight reductions comparable to those reported here following treatment with a DPP-4 inhibitor (mean −1.58 kg at 12 months) were also seen in a previous analysis of a US database, in which patients achieved a mean weight change from baseline of −1.26 kg at 12 months [42]. Previous studies have demonstrated the add-on benefit of SGLT-2 inhibitors by promoting weight loss via increased glucose excretion [43–46]. The weight reductions seen in this analysis following initiation with an SGLT-2 inhibitor were higher than those observed in clinical trials (mean weight reduction at 18 months of 4.2 kg versus 2.2–3.4 kg) [43] and continued at each time point up to 18 months, which may reflect a greater likelihood of treatment persistence in those who lose weight. Patients who received an SGLT-2 inhibitor-based treatment generally had a higher weight at baseline, suggesting selection of this therapy in more obese patients due to its established effects on weight, consistent with NICE guidelines [4, 47]. Given the demonstrated correlation between baseline weight and weight loss, this may also partly account for the greater weight loss observed with SGLT-2 inhibitors in this analysis. Additionally, the low number of patients who had weight measurements at 18 months in the SGLT-2 inhibitors group could be considered a possible confounder. Perhaps expectedly, no overall change in weight was observed in patients included in the other group, as this group included both patients who received agents that are known to cause weight gain (including insulins and TZDs) and patients who received agents associated with weight loss (including GLP-1 receptor agonists). The weight changes presented in this analysis were adjusted for baseline values to allow comparisons between treatment groups. However, the effect of second-line treatment on weight has been associated with ethnicity, with Asian populations showing a greater response to agents such as DPP-4 inhibitors [48]. Despite information on ethnic groups not being available in the CPRD, the Asian population in the UK represents approximately 7% of the general population [49] and, thus, this population effect should not be considered a potential bias.

A pooled analysis of previous clinical and observational studies reported that treatment persistence in patients with T2D receiving oral anti-diabetic agents ranged from 41.0 to 81.1%, and treatment discontinuation estimates ranged from 9.9% to 60.1% [50]. In the current study, treatment persistence was highest in patients receiving treatment with metformin plus an SGLT-2 inhibitor at all time points, with 57.7% of patients continuing with the index treatment at 18 months. By comparison, 53.2% of patients who received metformin plus either an SU or a DPP-4 inhibitor and 47.2% of patients on other treatments continued with their index therapies, respectively. In line with this, a previous study has shown greater treatment persistence in patients receiving SGLT-2 inhibitors versus SU [51]. The higher rates of treatment persistence seen with an SGLT-2 inhibitor may be associated with the steady decrease in HbA1c levels and weight reductions and reflect increased patient satisfaction with these therapies. Although the reasons for patient discontinuation, switching or intensification of therapy were not reliably recorded in the UK CPRD, and information on adherence to medication and completion of each course of treatment were limited to the available records, in clinical practice common reasons for discontinuation or change of treatment include undesirable side effects (such as weight gain or risk of hypoglycaemia) and inefficacy of treatment requiring intensification or change of therapy [52].

The median time to initiation of second-line treatment did not differ substantially among the different treatment groups investigated, with patients initiating second-line therapy a median of 2.3 years after metformin initiation. Therapeutic inertia remains an issue in clinical practice, with a previous analysis of the UK CPRD database observing a delay of 1.6–2.9 years before second-line treatment intensification in patients with suboptimal glycaemic control (study cohort of 81,573 patients) [27]. Another analysis of the UK CPRD (*n* = 6710) observed that only 39.5% of patients had their treatment intensified within the year following metformin monotherapy treatment failure (HbA1c ≥ 7% [53 mmol/mol]), and 24% of patients had no evidence of treatment intensification throughout the ~4.3 years of the study [31]. In an analysis of a large US insurance claims database (*n* = 11,525), which included a cohort of patients with T2D and poor glycaemic control (HbA1c ≥ 8% [64 mmol/mol]), more than half (52%) of the patients did not receive treatment intensification within 12 months of treatment failure [53]. Another study using a US claims database that included patients with T2D identified a mean time to treatment intensification after HbA1c levels were above the target of over 700 days [54]. A recent systematic review that assessed data from patients with T2D from North America, Europe and Israel showed that the median time to treatment intensification after HbA1c levels were above target was 0.3–2.7 years and 1.3–4.9 years, in patients receiving one or two oral anti-diabetic drugs respectively [28].

As an observational study, this analysis had a number of limitations. Firstly, as it aimed to represent a population on-treatment, patients at each time point who discontinued, switched or intensified the index second-line treatment, as well as those who did not have HbA1c levels or body weight data available, were censored from the study. The number of patients evaluated for changes in HbA1c levels and weight were, therefore, smaller at later time points. This is particularly noticeable for the analysis at 18 months, which may limit the generalisability of the results. Secondly, only patients whose first-line therapy was metformin monotherapy were included. Although these patients accounted for the majority of those identified initiating a second-line therapy in the CPRD, consistent with guidelines and clinical practice [4, 6], this patient population may limit the generalisability of these findings to patients who receive a different first-line therapy. Thirdly, consideration should be given to the limited use of some of the more recent glucose-lowering therapies, such as DPP-4 inhibitors and SGLT-2 inhibitors, which have not been used as widely, as is the case for older, more established therapies. Moreover, these more recent therapies were not recommended by NICE guidelines as a treatment choice for treatment intensification after metformin until December 2015, close to the cut-off date for inclusion in this study. Consequently, fewer patients were exposed to these agents, limiting the analyses and interpretations of these results. And fourthly, given the low number of patients identified who received therapy combinations other than metformin plus an SU, a DPP-4 inhibitor or an SGLT-2 inhibitor or received second-line therapy without metformin, these patients were combined into the other treatment group, which prevented reliable analysis of clinical outcomes for each individual treatment strategies.

In addition to the above, observational studies using databases such as the UK CPRD inherently have a number of limitations, including potential issues relating to the quality of the data for key clinical measurements and possible misclassification of exposure and outcome. Hypoglycaemia events were not reliably captured in the UK CPRD, which prevents the assessment of risk of hypoglycaemia for second-line therapy in this analysis. The reports for events such as diabetic ketoacidosis or urinary tract infections are also limited and, therefore, not reported in this analysis. These safety events have been formally evaluated in other post-authorisation safety studies specifically designed to that end, and their incidence evaluated in an independent meta-analysis [55] and in clinical practice [56]. In addition, there is the possibility of confounding by indication, as patients were not randomised to receive the treatments; rather, treatments were selected by the physician based on clinical judgement according to specific patient characteristics. This non-random selection resulted in the differences between the baseline characteristics of the treatment groups. While multivariate regression was undertaken to adjust for the measured differences, the possibility of residual confounding contributing to the findings remains.

## Conclusions

In this population-based cohort, all second-line therapies initiated after metformin monotherapy improved glycaemic control, with the greatest HbA1c reductions seen in patients with higher baseline HbA1c levels. In addition, significant reductions in weight were observed for patients treated with metformin plus either a DPP-4 inhibitor or an SGLT-2 inhibitor. The lowest incidence of second-line therapy change was seen in patients treated with metformin plus an SGLT-2 inhibitor. Patients treated with metformin plus an SGLT-2 inhibitor were most likely to achieve a composite of HbA1c reduction ≥ 0.5% (5.5 mmol/mol), weight reduction ≥ 2 kg and continued treatment at 18 months. This highlights the potential benefits of treating patients with more recent agents, including SGLT-2 inhibitors. The ongoing, randomised, pragmatic trial (DECIDE; NCT02616666) aims to evaluate the comparative effectiveness between the SGLT-2 inhibitor dapagliflozin and standard of care over 2 years in patients with T2D, and should provide further data on this therapeutic class [57].

## Additional files

Additional file 1: **Figure S1.** Study attrition. (TIF 53 kb)

Additional file 2: **Figure S2.** Relationship between change in weight at 18 months and baseline weight according to second-line treatment. Scatter plots of baseline (index) weight against change in weight at 18 months in patients on metformin plus either an SU (**a**), a DPP-4 inhibitor, (**b**) an SGLT-2 inhibitor (**c**), or other therapies (**d**). Regression lines are shown as grey dashed lines, and their corresponding gradient slopes (*m*) were calculated for baseline weight. (TIF 3255 kb)

## Abbreviations

BMI: Body mass index
CPRD: Clinical Practice Research Datalink
DPP-4: Dipeptidyl peptidase-4
GLP-1: Glucagon-like peptide-1
IQR: Interquartile range
MET: Metformin
NICE: National Institute for Health and Care Excellence
SD: Standard deviation
SE: Standard error
SGLT-2: Sodium–glucose cotransporter 2
SU: Sulfonylurea
T2D: Type 2 diabetes
TZD: Thiazolidinedione
