# Supplementary figures and images for: Changes in HbA1c and weight, and treatment persistence, over the 18 months following initiation of second-line therapy in patients with type 2 diabetes: results from the United Kingdom Clinical Practice Research Datalink
Source: BMC Med. 2018 Jul 16;16:116. doi: 10.1186/s12916-018-1085-8 (PMC6047134; doi:10.1186/s12916-018-1085-8)

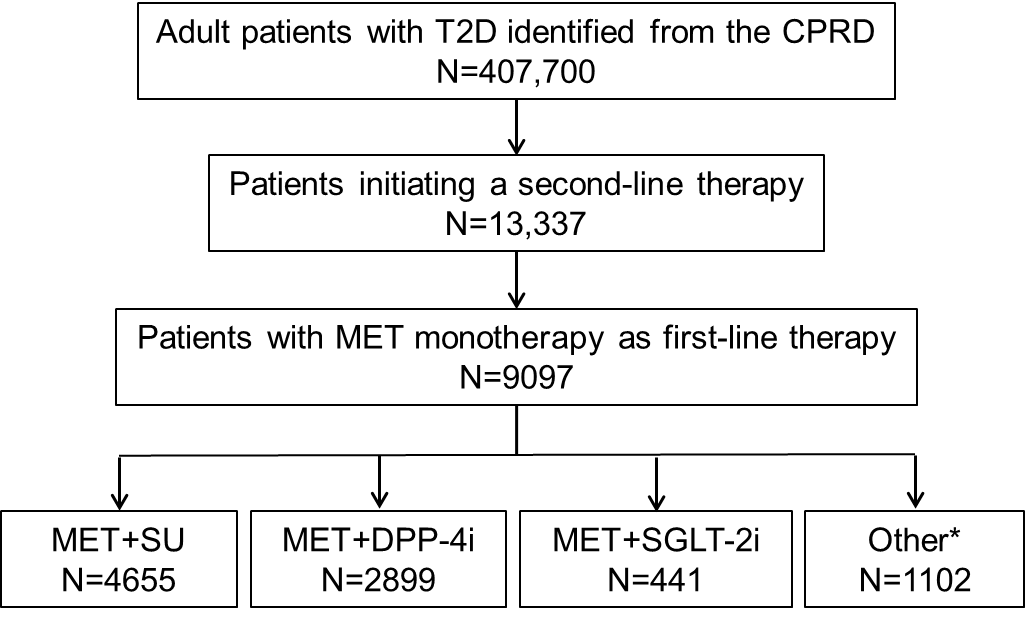

Supplement: Supplementary file 1 — Figure S1. Study attrition. (TIF 53 kb) [file 12916_2018_1085_MOESM1_ESM.tif]

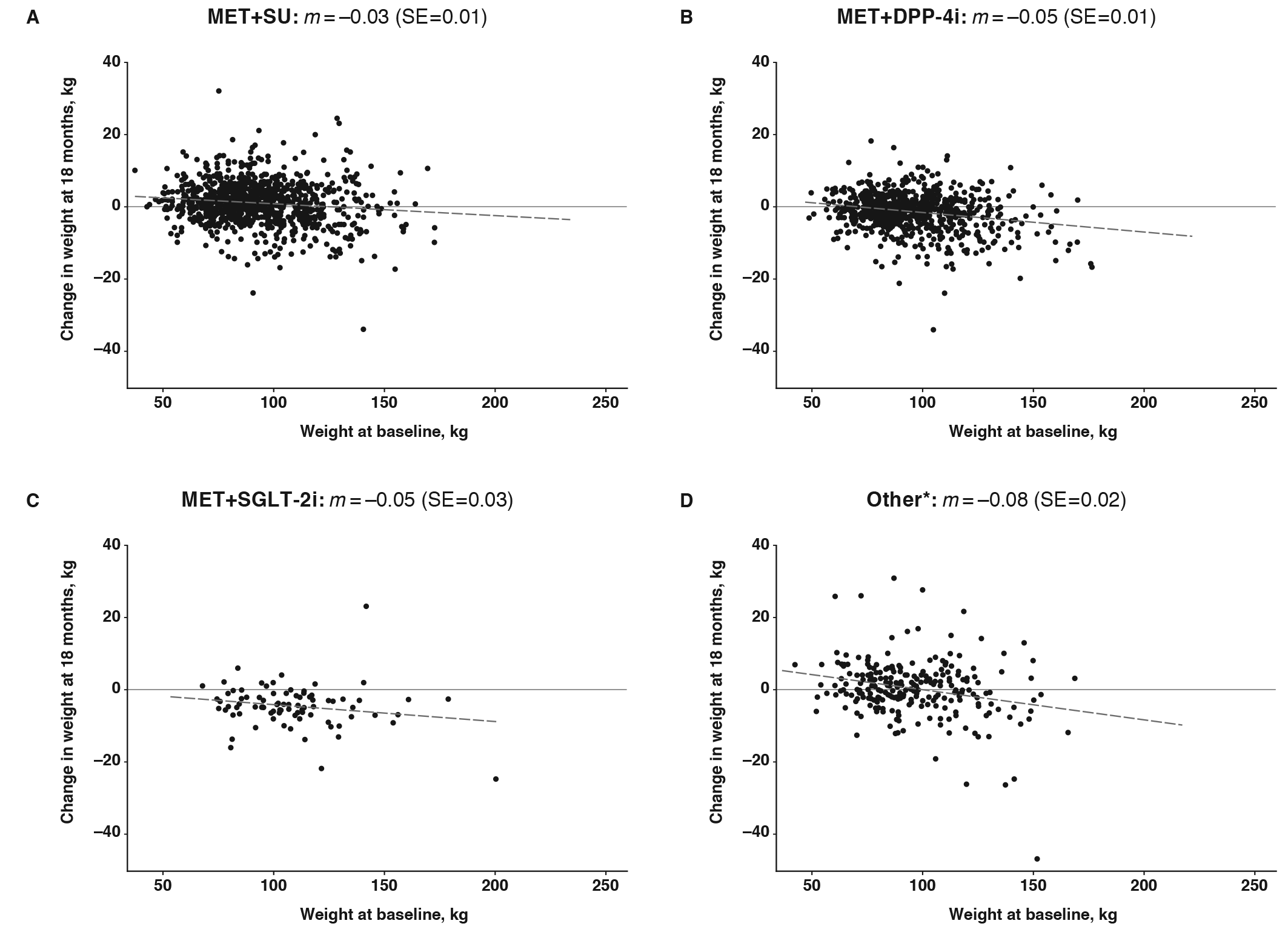

Supplement: Supplementary file 2 — Figure S2. Relationship between change in weight at 18 months and baseline weight according to second-line treatment. Scatter plots of baseline (index) weight against change in weight at 18 months in patients on metformin plus either an SU (a), a DPP-4 inhibitor, (b) an SGLT-2 inhibitor (c), or other therapies (d). Regression lines are shown as grey dashed lines, and their corresponding gradient slopes (m) were calculated for baseline weight. (TIF 3255 kb) [file 12916_2018_1085_MOESM2_ESM.tif]
